# Long fragments achieve lower base quality in Illumina paired-end sequencing

**DOI:** 10.1038/s41598-019-39076-7

**Published:** 2019-02-27

**Authors:** Ge Tan, Lennart Opitz, Ralph Schlapbach, Hubert Rehrauer

**Affiliations:** Functional Genomics Center Zurich, ETH Zurich/University of Zurich, Zurich, Switzerland

## Abstract

Illumina’s technology provides high quality reads of DNA fragments with error rates below 1/1000 per base. Sequencing runs typically generate millions of reads in which the vast majority of the reads has an average error rate below 1/1000. However, some paired-end sequencing data show the presence of a subpopulation of reads where the second read (R2) has lower average qualities. We show that the fragment length is a major driver of increased error rates in the R2 reads. Fragments above 500 nt tend to yield lower base qualities and higher error rates than shorter fragments. We use publicly available Illumina data to demonstrate that the fragment length dependency of the R2 read qualities exists in various library protocols, in different labs and using different sequencer models. Our finding extends the understanding of the Illumina read quality and has implications on error models for Illumina reads. It also sheds a light on the importance of controlling the fragment size during library preparation.

## Introduction

Next generation sequencing (NGS) has become one of the most widely used technologies in biomedical research with a rapid development of new applications. Due to its efficiency and low financial cost, NGS has substantially improved many projects, including *de novo* genome assembly sequencing, resequencing, transcriptome profiling, sequence variants detection, epigenome profiling and chromatin interaction. Illumina platforms represent, by far, the most widely used technology for generating short reads. Current Illumina platforms produce millions to billions of reads in a single run. Illumina reports base quality in terms of a Phred-like quality score and claims the “vast majority of bases scoring Q30 and above” (https://www.illumina.com/science/education/sequencing-quality-scores.html), where Q30 suggests an average per base error rate of 1/1000^1–3^. The high quality of Illumina reads is among the reasons for Illumina’s success, and can be verified readily by analyzing the wealth of Illumina data deposited in public repositories. However, we made the intriguing observation that in Illumina paired-end sequencing the quality of the second read can be low and have a wide spread within a sample. It can be expected that the average quality of the second read is lower, however, the magnitude of the quality drop relative to the first read can vary a lot between reads of the same library in the same run. As a consequence, a sizeable population of second reads with average quality well below Q30 may be observed even if all first reads have high quality. The size of this fraction of low quality reads can vary largely between runs, and even within the same run it may vary between multiplexed libraries of the same pool. Currently existing error models of Illumina reads do not provide an explanation for this phenomenon.

Previous studies have analyzed the characteristics of Illumina errors, showing that sequencing errors are not completely random but exhibit systematic behaviors. The dominant error type of introduced errors are substitutions with very low rates of insertions and deletions and the read quality tends to be lower towards the end^4^. Certain preferences of substitutions errors have been also observed; Dohm *et al*. and Nakamura *et al*. reported wrong bases tend to be preceded by base G^4,5^. The frequency of the most frequent A to C conversion can be ten- to eleven-fold over the least frequent conversion from C to G^4^. Nakamura *et al*. also proposed that inverted repeats and GGC sequences patterns are the major causes of sequence-specific errors^5^. Meacham *et al*. observed the systematic error of frequent sequence motif GGT due to substitution from GGG motif^6^. A dependency of the quality on the base position in the read, as well as strand-specific bias, was also reported^7^. Huptas *et al*. observed that the larger the GC content and the larger the insert size, the fewer read pairs survive read quality filtering^8^. Analyses of multiple datasets from several sequencers revealed the motifs that induce read errors are platform-dependent^9^. However, none of the above findings explains the phenomenon of low read quality in subpopulations of the second read (R2) even when the first read (R1) has high quality. Although Soumitra *et al*. proposed a k-mer approach to filter out the error-containing reads at a high precision rate^10^ a clear understanding of the source of errors from this paired-end sequencing is desired.

In this manuscript, we demonstrate that fragment length impacts the error rates, especially of the second read, explains the observation of populations of R2 reads with low average quality. For this we determined fragment lengths by read alignment and stratified reads according to the fragment length. Based on the alignments we computed base mismatch rates as a measure of base quality and evaluated how the fragment length influences the base mismatch rates of the R1 and the R2 reads. We performed the analyses using sets of publicly available Illumina read data that we collected from Illumina BaseSpace and the short read archive (SRA). Our analysis covers various sequencer models as well as library protocols. In the subsequent sections we report our results, discuss them, and describe our methods.

## Results

### Quality of the second read in paired-end sequencing

We surveyed publicly available paired-end sequencing data downloaded from Illumina BaseSpace and SRA that cover various library protocols and sequencer models (see Table 1). The identifier of the study is either the BaseSpace project name or the SRA project ID. In total, our dataset comprises 138 sequenced libraries from 27 different projects. In order to simplify read alignment, we included only sequencing data from human or mouse samples.

**Table 1:** Summary of investigated datasets from BaseSpace and SRA.

Study	Protocol	Sequencer Read length	Species	#Samples
NovaSeq S1 Xp: Nextera DNA Flex (Replicates of NA12878)	WGS	NovaSeq 6000 (2*151 bp)	Homo sapiens	1/4
HiSeqX: Nextera DNA Flex (replicates of Coriell Trio Samples)	WGS	HiSeqX (2*151 bp)	Homo sapiens	1/24
HiSeq 4000: TruSeq PCR-Free (NA12878)	WGS	HiSeq 4000 (2*151 bp)	Homo sapiens	1/6
HiSeq 2500: TruSeq PCR-Free DNA 2 × 251 (NA12878)	WGS	HiSeq 2500 (2*251 bp)	Homo sapiens	1/2
HiSeq X: TruSeq PCR-Free and Nano (UDI Replicates of NA12878 + /- Free Adapter Blocking Reagent)	WGS	HiSeqX (2*151 bp)	Homo sapiens	64/64
NovaSeq S2: TruSeq PCR-Free 350 (Replicates of NA12878)	WGS	NovaSeq 6000 (2*151 bp)	Homo sapiens	12/12
HiSeq 4000: TruSeq PCR-Free and Nano (350 bp to 550 bp insert size)	WGS	HiSeq 4000 (2*151 bp)	Homo sapiens	8/8
HiSeq 4000: RNA-Seq 64-plex (MAQC HBRR and UHRR)	RNA-Seq	HiSeq 4000 (2*76 bp)	Homo sapiens	1/64
HiSeq 2500: TruSeq Stranded mRNA LT (SEQC: UHRR & Brain)	RNA-Seq	HiSeq 2500 (2*75 bp)	Homo sapiens	1/4
NovaSeq S2: TruSeq Exome (96 replicates of NA12878)	WES	NovaSeq 6000 (2*76 bp)	Homo sapiens	1/96
HiSeq 4000: TruSeq Exome (12plex replicates of NA12878)	WES	HiSeq 4000 (2*76 bp)	Homo sapiens	1/48
HiSeq 2500: TruSeq Rapid Exome (12 replicates of NA12878)	WES	HiSeq 2500 (2*101 bp)	Homo sapiens	1/12
SRP066955	RNA-Seq	HiSeq 4000 (2*50 bp)	Mus musculus	4/4
SRP139105	RNA-Seq	HiSeq 4000 (2*151 bp)	Mus musculus	4/16
SRP137053	RNA-Seq	HiSeq 4000 (2*126 bp)	Mus musculus	4/16
SRP132952	RNA-Seq	NovaSeq 6000 (2*101 bp)	Mus musculus	4/12
SRP124637	RNA-Seq	NovaSeq 6000 (2*150 bp)	Mus musculus	2/15
SRP143964	RNA-Seq	HiSeq 2500 (2*150 bp)	Mus musculus	3/18
SRP142426	RNA-Seq	HiSeqX (2*150 bp)	Mus musculus	4/6
SRP136620	RNA-Seq	HiSeqX (2*150 bp)	Mus musculus	4/6
SRP143392	RNA-Seq	HiSeq 2500 (2*101 bp)	Mus musculus	4/9
SRP124981	WGS	HiSeq 4000 (2*150 bp)	Mus musculus	1/32
SRP072373	WGS	HiSeq 4000 (2*100 bp)	Mus musculus	1/1
ERP016331	WGS	HiSeq 2500 (2*151 bp)	Mus musculus	2/487
SRP145593	WGS	HiSeqX (2*150 bp)	Mus musculus	2/9
SRP132517	RNA-Seq	NextSeq 500 (2*75 bp)	Mus musculus	3/10
SRP129935	RNA-Seq	NextSeq 500 (2*76 bp)	Mus musculus	3/14

**Figure 1:**
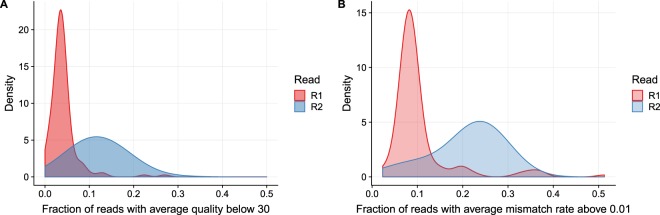
We plot the fraction of low quality reads in the 138 samples analyzed in our study. Across all samples the R2 reads harbor more low quality reads than the R1 reads. We plot two alternative definitions of ‘low quality’. Reads are called low quality if (**A**) the average Phred score is below 30, or (**B**) the average mismatch rate of the aligned bases is above 0.01. Both plots show that the R2 reads harbor more low quality reads and that the fraction of low quality reads is more variable across samples.

As a first step of quality evaluation, we quantify the fraction of low quality reads in samples. Figure 1 shows that, generally, the R2 reads tend to host a larger fraction of low quality reads. Figure 1A is based on the Phred-scaled base quality values generated by Illumina’s base-calling software and shows the fraction of reads that has a mean Phred score below 30. Figure 1B is based on the mismatch rates after read alignment and shows the fraction of reads that has an average base mismatch rate above 1/100. Both measures give a similar result and this corresponds with the finding by Sunyoung *et al*. who reported that both measures are well correlated^2^. The plots show that the R2 reads tend to have larger fractions of low quality reads than the R1 reads. The remarkable characteristic is that the fraction of low quality R2 reads is apparently strongly variable between samples and leads to a broad density distribution while the density distribution for the R1 read is narrow. This points to an additional error-driving factor that influences only the R2 read.

### Mismatch rates as read quality measure

In the rest of the manuscript, we use exclusively the mismatch rate after paired-end alignment as a quality measure of a read. We only consider properly paired alignments and we evaluate the mismatch rate separately for the R1 and the R2 read. Our subsequent analysis performs only intra-sample comparisons where the mismatch rates of the R2 and R1 reads are related to each other. With that approach we eliminate potential sample-to-sample differences that are caused by alignment errors or by genotype differences of individual samples from the reference genome. In our analysis, we call a read a ‘low quality read’ if its average mismatch rate is above 1%, independent of the read length.

### Fragment lengths and mismatch rates

**Figure 2:**
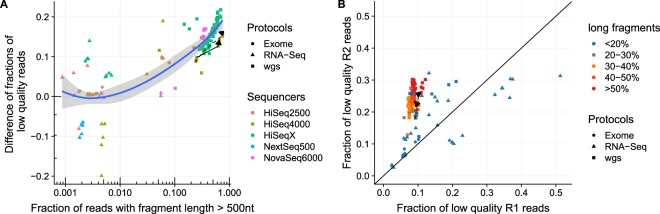
Increase of R2 low quality reads as a function of the content of long fragments. In (**A**) we plot for individual samples the difference in low quality read content among the R2 and the R1 reads versus the content of long fragments. The plot shows that the more long fragments a samples has the more prevalent are low quality reads among the R2 reads. In (**B**) we directly compare the fraction of low quality reads in R2 and R1 and color-code the content long fragments. Low quality reads are defined as reads having a mismatch rate above 0.01 in the bases after alignment. The plotted samples have been generated using various protocols on various sequencers in various labs. The dashed lines connect three samples each that have been processed identically except with an increasing targeted fragment length.

When investigating what drives the magnitude of the quality drop of the R2 reads, we found that the fragment length distributions of the libraries have a major impact. Specifically, the fraction of low quality R2 reads is related to the fraction of long fragments in the library. We consider a fragment as ‘long’ if the size of the fragment is above 500 nt. As Fig. 2 shows, the proportion of low quality R2 reads additional base mismatches in the R2 read relative to the R1 read is correlated with the amount of fragments that have a length above 500 bp. Figure 2A shows that in libraries where the proportion of long fragments is below 20%, the R1 and the R2 reads tend to have similar proportions of low quality reads. If libraries have increased proportions of long fragments (>30%), the R2 reads contain more low quality reads than the R1 reads. Figure 2A with linear scale in X-axis is shown in Supplementary Fig. S1. Figure 2B visualizes the absolute fractions of low quality reads in R1 and R2 in a scatter plot and indicates for each sample the fraction of long fragments by the coloring of the plotting symbols. Additionally we used Spearman’s correlation test to verify this hypothesis. The results are shown in Table 2. The proportion of low quality reads in the R1 is not correlated with the content of long fragments but the proportion of low quality R2 reads is. The highest correlation score is obtained for the difference of low quality R2 minus low quality R1 reads.

**Table 2:** Correlation tests of mismatch rate and content of long fragments.

proportion of long fragments vs	Correlation coefficient	p-value	significant with alpha = 0.05
proportion of low quality R1 reads	−0.019	0.825	not significant
proportion of low quality R2 reads	0.590	<2.2e-16	significant
R2 - R1 difference of low quality read proportions	0.841	<2.2e-16	significant

As the plots in Fig. 2 show, this characteristic is observed for all library types and all sequencer models analyzed. Notably, the data analyzed includes two experiments where the starting material, library preparation and sequencing was identical. The only difference in those samples is the targeted fragment length (350, 450 and 550 nt, respectively). The results of these samples are highlighted in Fig. 2 by dashed lines. Figure 2B shows that for those samples the fraction of low quality R2 reads strictly increases with the fraction of long fragments. The trend observed in those samples corroborates that the low quality R2 reads are driven by fragment length and not by tissue source, library type or sequencer model.

On a side note, Fig. 2B also shows that for 11 RNA-Seq experiments the R1 read has a higher fraction of low quality reads than the R2 reads, which comes as an unexpected result at first glance. However, it is known that Illumina’s stranded RNA-Seq protocols lead to lower quality bases at the R1 read start (5′-end of the R1 read, 3′-end of the fragment)^11^. A closer inspection revealed that in those four samples this 3′-end low quality effect is very strong, actually stronger than the R2 read quality drop (Supplementary Fig. S4A). Since the fragments are longer than the read length the low quality 3′-end of the cDNA only affects the R1 read but not the R2 read.

To ensure this finding is not due to the artefacts of the Bowtie2^12^ aligner, we applied another aligner BWA^13^. As shown in Supplementary Fig. S2, the same behaviour is observed for BWA. In addition, to make sure the alignment accuracy is not largely impaired by the low quality read bases, we performed a trimming of low quality, leading and trailing bases using Trimmomatic^14^. Even with aggressive trimming options, in Supplementary Fig. S3, we can still see the same effect.

**Figure 3:**
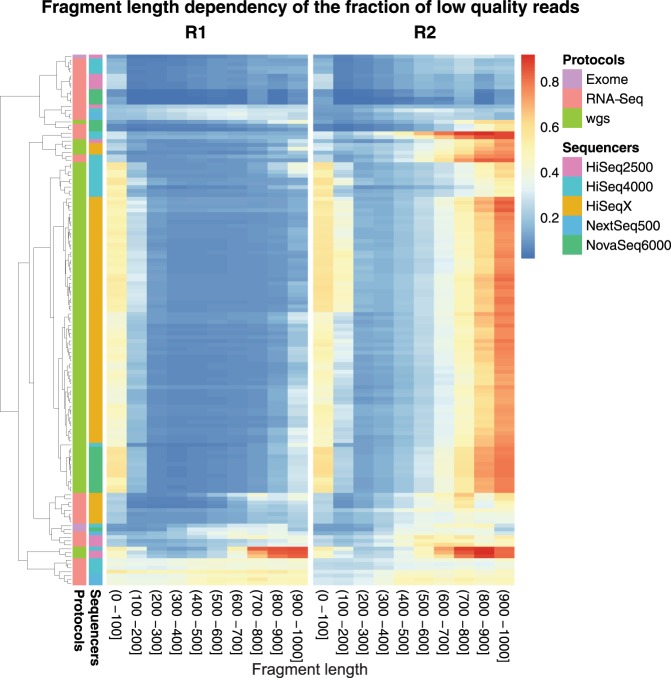
Fraction of low quality reads visualized as heatmap. The Y-axis represents the individual samples color-coded by library preparation protocol and sequencer model. For each samples the reads are stratified by fragment length and the X-axis indicates the stratum. The R1 reads (left) have a low prevalence of low quality reads while the R2 reads exhibit an increase of low quality reads for fragments longer than 500 nt.

In order to assess the details of the dependency of base and read quality on the fragment length, we stratified the reads according to fragment length and analyzed the strata separately. Figure 3 shows the fraction of low quality reads for different fragment length strata. The plot confirms that for the R1 read the fraction of low quality reads has no strong systematic dependency on the fragment length. Reads are reliable for fragments ranging from 200 to 1000 bp. In contrast to this, the fraction of low quality reads increases clearly for fragments in the range of 500 to 600 bp and above. Again the plot shows that this trend is observable for different sequencer models and library types.

### Dependency of base quality on base position

**Figure 4:**
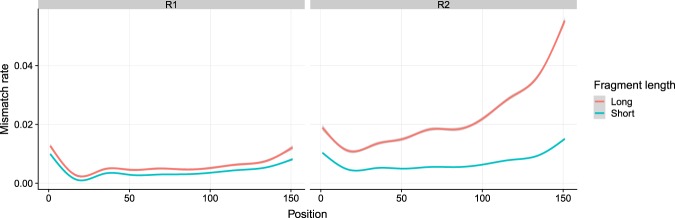
Position specific averaged mismatch rate of short and long fragments in R1 and R2 for all datasets. In R1, the short and long fragments exhibit similar position specific mismatch rate, while in R2, the long fragments lead to much higher mismatch rate than short fragments which even increases towards the end of the read. The smoothing line was generated by “gam” and the confidence interval is 0.99.

We additionally verified the positions of the low quality bases and characterized the distribution of low quality positions. It is known that Illumina’s sequencing by synthesis leads to a quality decrease, as more and more bases are sequenced. This is caused by the fact that single molecules in a cluster go out of sync^4^. At every sequencing step individual molecules of a cluster may fail to get a newly synthesized base. If the number of erroneously synthesized molecules in a cluster increases the sequencer detects a mixed signal which can lead to base calling errors. In Fig. 4, we plot the average mismatch rates per base position for short fragments (200–400 bp) and long fragments (600–1000 bp) in R1 and R2 reads separately. The left panel confirms the known behavior of decreasing quality towards the end, but also shows that the R1 quality of long fragments is very close to the quality of short fragments. The right panel, however, shows that the R2 reads follows a different pattern. The early bases in the long fragments have a mismatch rate that is clearly above the mismatch rate observed for short fragments. Interestingly the length-dependent increase in the mismatch rate for the long fragments is also more pronounced than for the short fragments.

## Discussion

This is the first time that the impact of the fragment length on the base qualities of Illumina reads has been systematically investigated and quantified. Previous research has looked at other various factors like sequence context, base position in read, *etc*., but none of these explains the widely observed lower quality of the R2 reads that happens only in a sub-populations of the reads from the same sample on the same sequencing run. Our analysis identifies the fragment length as the major cause for this higher risk of low quality of an Illumina R2 read relative to the corresponding R1 read of the same fragment. We find that if libraries have higher fractions of long fragments then the reported Phred scores tend to be lower and the mismatch rates in paired-end alignment tend to be higher. Overall our analysis confirms that the Phred qualities are good representatives of the actual base quality. We also find that sequencing quality is optimal if libraries are prepared according to Illumina’s specifications with short fragment lengths like 250 bp for RNA-Seq and 350 bp for DNA-seq. Additionally, we can confirm that the presence of low quality reads from long fragments does not affect the high quality of reads achieved for the shorter fragments in the same library that are in the range of 200 to 400 bp.

Our conclusions do not rely on an analysis of the Phred-scale qualities computed by the Illumina software but rather on the base mismatches after paired-end alignment. By that, our results are independent of proprietary software and we can guarantee that our computations do not consider the sequencer model in any form. We only make statements on the differences of base mismatch rates between short and long fragments and the corresponding differences between the first and the second read. With this approach, mismatches in the alignment that are caused by genotype differences and misalignments do cancel out. This approach also avoids that our findings are confounded by other factors that influence the likelihood of sequencing errors. Examples of such factors are sequence context^5,15^ or GC content^8^ of the input material. We agree with those authors that these factors do impact read quality, but a discussion of those factors is beyond the scope of this manuscript and we refer the reader to the cited literature. Our study acknowledges that the mismatch rate varies between samples in different studies due to the potential presence of such factors, but the observed increase of the mismatch rate in the R2 read of long fragments is consistently observed in all studies. Since our study suggests that the optimal quality of Illumina reads is achieved if the input library has only short fragments, the protocols that intrinsically generate short fragments are less affected. These protocols include, e.g., whole exome sequencing protocols where the enrichment of exonic fragments uses sheared input DNA in the range of 150–200 nt. For all samples investigated, we observed a high mapping rate. This suggests that in applications where aligned reads are finally counted and only the read counts are used, the reads from the long fragments are still valuable. The fragment length effect on quality limits the choices of fragment lengths in *de novo* sequencing projects. Longer fragments would help the assembly but are not advisable due to the increased error rate. While reads from long fragments can be filtered out in resequencing projects based on Phred quality, fragment length and base mismatches. Such an approach is not possible in a *de novo* setting where read alignments are not available.

Based on our results a fragment length filter as a preprocessing step of variant calling might be useful in situations where low frequency mutations are searched in heterogeneous cell populations, or where variants are called from low coverage sequencing data. The results also give indications that the modelling of Illumina errors (for a review of read error modelling see^16^) can be improved by including fragment length as a factor. However, the fact that the R1 reads are high quality independent of the fragment-length suggests that none of the steps until sequencing the R1 read is affected. This implies that cluster amplification works equally well for short and long fragments.

## Methods

### Data processing and mapping

Reads were preprocessed by Trimmomatic^14^ (ILLUMINACLIP:adapters.fa:1:20:7:5:true MINLEN:20) in order to remove adapters from the 3′-end. Subsequently reads were aligned with Bowtie2^12^ (–no-unal -D 20 -R 3 -N 1 -L 20 -i S,1,0.50–maxins 1000–minins 0). We chose to be error-tolerant in order to map as many reads as possible. The sensitive options will also align the reads with many errors. Being stringent would have implied that low quality reads were discarded, which would have biased our result. The parameter choices will report concordant paired-end alignments only if the fragment length is 1000 bp or below. We found that this does not represent a limitation since in all libraries the number of fragments with larger size were very low. In fact so low that the we considered them too few in order to draw conclusions. SRA and BaseSpace were aligned against mouse (GRCm38.p5) and human (GRCh38.p10) references, respectively. The whole-genome sequencing reads were aligned to reference genome, while RNA-seq reads were aligned against the corresponding set of transcripts downloaded from Ensembl, extended with 125 bp long poly-A sequences to each transcript. We chose transcriptome alignment in order to be able to use the same aligner as for the DNA samples and still have a good estimate of fragment length. This choice implies that reads that come from splice variants not represented in the transcriptome will be omitted. In order to minimize errors caused by alignment errors, we look only at the primary alignments. And since we inspect the dependency on fragment length we only look at properly paired reads. For all others we would not know the fragment lengths. Aligned BAM files were processed with samtools^17^ to produce MD tags and identify the bases with mismatches. All computations and plots were computed with R scripts.

In order to further strengthen the result, we applied a trimming of low quality bases using Trimmomatic with the options (SLIDINGWINDOW:4:20 LEADING:20 TRAILING:20 MINLEN:20) followed by Bowtie2 alignment. Those results together with the results generated when using BWA mem as aligner are shown as additional results in the Supplementary Material.

## Supplementary information

Supplementary Material

## Data Availability

All data used in this study is publicly available at the Short Read Archive and Illumina Basespace. Identifiers are indicated in the manuscript.
