# Supplementary material for: Long fragments achieve lower base quality in Illumina paired-end sequencing
Source: Sci Rep. 2019 Feb 27;9:2856. doi: 10.1038/s41598-019-39076-7 (PMC6393434; doi:10.1038/s41598-019-39076-7)
Supplement: Supplementary file 1 — Supplementary Material [file 41598_2019_39076_MOESM1_ESM.docx]

Supplementary material to

Long fragments achieve lower base quality in Illumina paired-end sequencing

Ge Tan^1^, Lennart Opitz^1^, Ralph Schlapbach^1^, Hubert Rehrauer^1^*

^1^Functional Genomics Center Zurich, ETH Zurich/University of Zurich, Zurich, Switzerland

*To whom correspondence should be addressed. Tel: +41 44 635 39 24; Fax: +41 44 635 39 22; Email: [hubert.rehrauer@fgcz.ethz.ch](mailto:hubert.rehrauer@fgcz.ethz.ch)

# Supplementary Figure 1


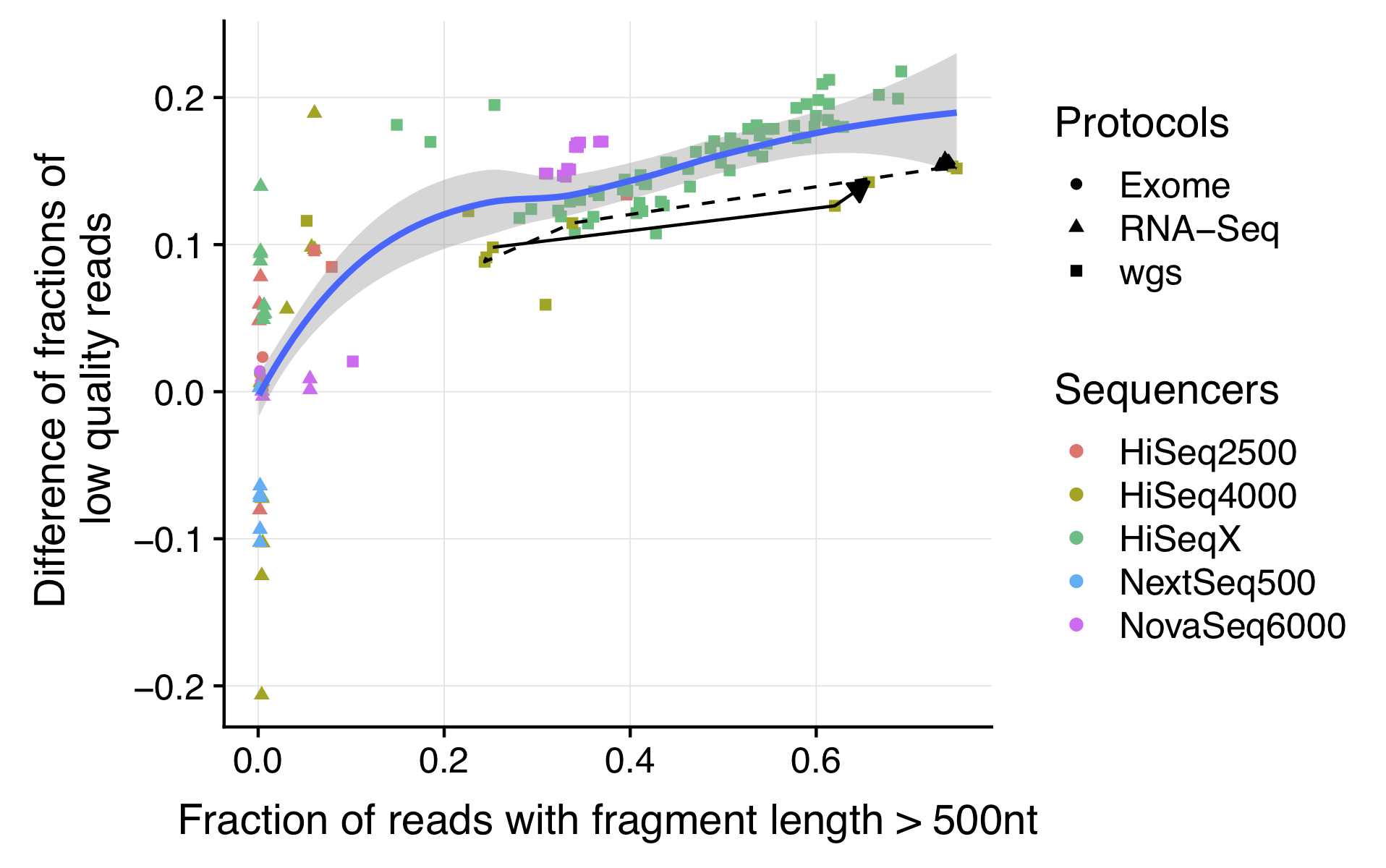


The main Figure 2B now shown with linear scaling of the X-axis instead of logarithmic scaling.

# Supplementary Figure 2


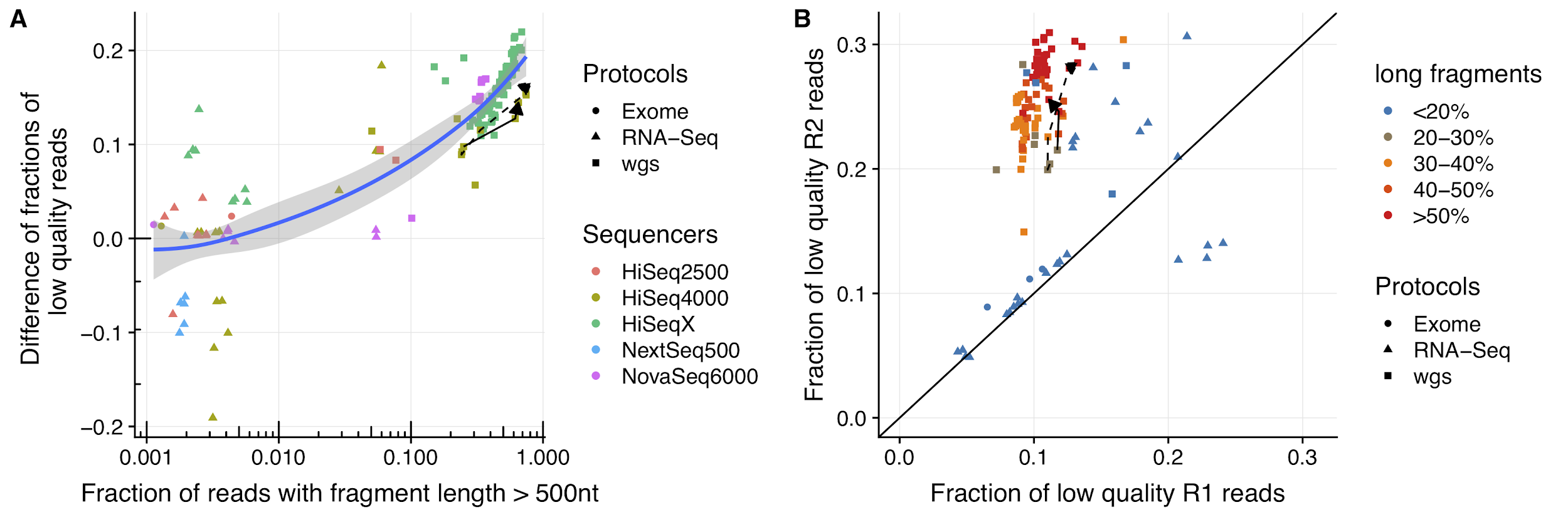


This figure corresponds to the main Figure 2 but is now generated using the results from the BWA aligner.

# Supplementary Figure 3


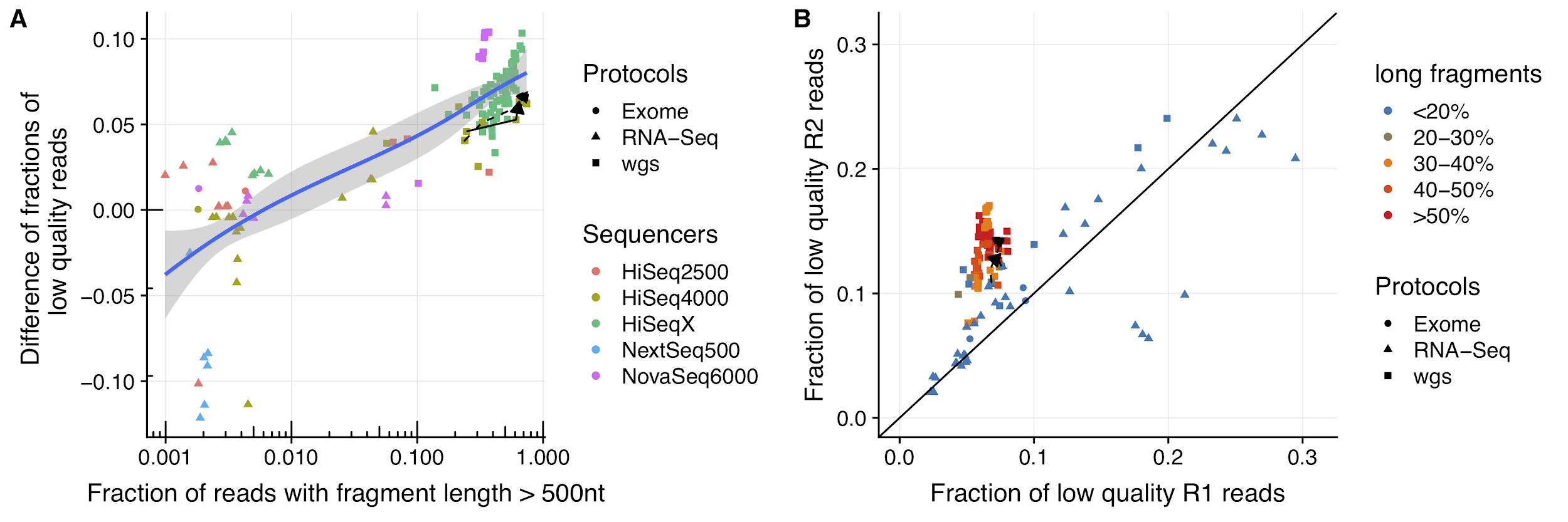


This figure corresponds to the main Figure 2 but is now generated using an aggressive trimming of bases with low Phred-scale quality at the beginning and end of the reads, and subsequent Bowtie2 alignment. The aggressive trimming of low quality bases increases the average base quality, especially of the R2 reads. As a consequence the fragment length effect on the R2 mismatch rates is reduced but still present.

# Supplementary Figure 4


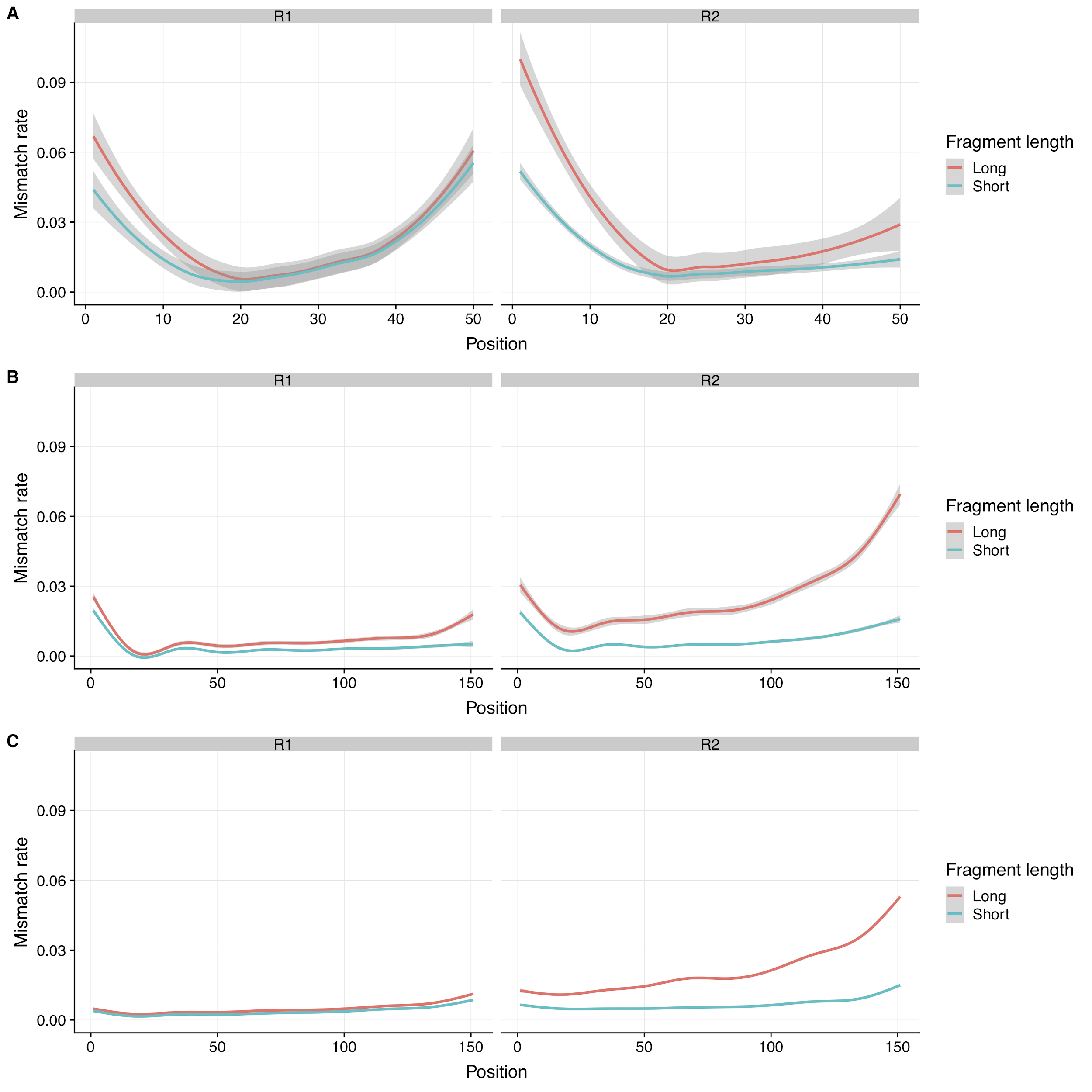


Position specific averaged mismatch rate of short and long fragments in R1 and R2 for stratified datasets. (A) RNA-Seq datasets with read length shorter than 50 nt, where the R1 has more low quality reads than the R2. All of these samples exhibit a very high base mismatch rate at the 5’- of the R1 reads. This represents an additional effect that outplays the general tendency of lower quality of the R2 bases. (B) RNA-Seq datasets with read length longer than 100 nt. (C) The datasets of other protocols. The smoothing line was generated by "gam" and the confidence interval is 0.99.
